# Supplementary material for: A Ca2+-regulated deAMPylation switch in human and bacterial FIC proteins
Source: Nat Commun. 2019 Mar 8;10:1142. doi: 10.1038/s41467-019-09023-1 (PMC6408439; doi:10.1038/s41467-019-09023-1)
Supplement: Supplementary file 1 — Supplementary information file [file 41467_2019_9023_MOESM1_ESM.pdf]

A Ca^{2+}-regulated deAMPylation switch in human and bacterial FIC proteins.

S. Veyron et al.

Supplementary Discussion.

Analysis of the deAMPylation catalytic mechanism of EfFIC.

We discuss below the mechanism of deAMPylation of EfFIC. First, it should be noted that FIC active sites do not ressemble the active site of *Legionella* de-AMPylase SidD, which has a canonical phosphatase fold [1] or the de-AMPylation domain of *E. coli* glutamine synthase adenylyl transferase [2], hence are not expected to share catalytic features with these enzymes. From a general enzymology perspective, two major mechanisms of (phospho)ester bond hydrolysis can be considered: anchimeric catalysis, which is assisted by the substrate (**Supplementary Figure 3A)** or conventional acido-basic catalysis **(Supplementary Figure 3B**). Both mechanisms involve four steps and share three chemical requirements: i) a proton attractor to increase the nucleophilic properties of the reactive oxygen (step 1) ii) a positively charged species, located in the neighborhood of the phosphate group to increase the susceptibility of the phosphorus to nucleophilic attack and stabilize the developing negative charge in the intermediate (steps 2 and 3), and iii) a proton donor, located close to the cleaved phosphor-ester bond to favor the production of the leaving group by giving up its proton (step 4). A major difference between the two scenarii is that nucleophilic attack is performed by the 2' hydroxyl of the ribose in the anchimeric mechanism, as described for a calcium-dependent phosphoinositide-specific phospholipase C from *Pseudomonas* [3], whereas it is achieved by the oxygen of an activated water molecule in the general acido-basic catalysis. The two mechanisms can be distinguished by at least two major features of the active site: i) anchimeric catalysis requires a proton attractor close to the ribose 2'OH, whereas the general acidic catalysis requires a proton attractor close to the nucleophilic water molecule, itself located close to the leaving phosphate group; ii) substrate-assisted catalysis involves the formation of a cycle between the phosphorus and the 2'OH of the AMP moiety of the substrate.

Considering our EfFIC^{WT}-AMP-Ca^{2+} structure as an acceptable mimic of the enzyme-AMPylated protein complex, EfFIC displays several important features: i) the AMP moiety is stabilized by multiple interactions with the active site, such that a large conformational change needed for cyclisation in the anchimeric reaction is unlikely ii) a water molecule coordinated by the conserved glutamate and Ca^{2+} ion can be readily positionned for in line nucleophilic attack by completing the heptahedral coordination of Ca^{2+} iii) no proton acceptor is situated closed to the 2'OH in the structures. Together, these observations impose a

mechanism based on acido-basic and electrostatic catalysis, as described in **Figures 5** and **S3B**.

Supplementary Methods

Crystallizations and structure determinations.

For crystals of EfFIC^{WT}- PO_4^{2}, the protein was prepared in a buffer containing 50 mM Tris pH 8.0, 100 mM NaCl, 1 mM $MgCl_2$, 1mM DTT and 10 mM NAD and concentrated to 10 mg/mL. Crystals were obtained in 0.16 M calcium acetate, 0.08M sodium cacodylate pH 6.5, 14.4 % (w/v) PEG 8000 and 20% (v/v) glycerol. Diffraction data were collected at beamline PROXIMA1 (SOLEIL synchrotron). The asymmetric unit contains 2 molecules, and EfFIC assembles as dimers in the crystal. The structure has been deposited with PDB entry code 6ER8.

For crystals of EfFIC^{WT}, the protein was prepared in a buffer containing 50 mM Tris pH 8.0, 100mM NaCl and 10mM ADP and concentrated to 13mg/mL. Crystals were obtained in 50mM Bicine pH 8.4 and 30% (w/v) PEG 2000 MME. Diffraction data were collected at beamline ID30A-3 (ESRF synchrotron). The asymmetric unit contains 4 molecules, and EfFIC assembles as tetramers related to the NmFICtetramer. The structure has been deposited with PDB entry code 5NV5.

For crystals of EfFIC^{WT}-AMP-Ca^{2+}, the protein was prepared in a buffer containing 50 mM Tris pH 8.0, 100 mM NaCl and 5 mM AMP and concentrated to 8 mg/mL. Crystals were obtained in 0.2 M calcium chloride, 0.1 M HEPES sodium salt pH 7.5 and 28 % (v/v) PEG 400. Diffraction data were collected at beamline ID29 (ESRF synchrotron). The asymmetric unit contains 2 molecules, and EfFIC assemble as dimers. The structure has been deposited with PDB entry code 6EP0.

For crystals of EfFIC^{WT}-ATPγS-Ca^{2+}, the protein was prepared in a buffer containing 20 mM Hepes pH 7.4, 200 mM NaCl, 5 mM $MgCl_2$ and 5 mM ATPγS and concentrated to 8 mg/mL. Crystals were obtained in 14.4 % w/v PEG 8,000, 20 % v/v glycerol, 80 mM MES pH 6.5 and 160 mM calcium acetate. Diffraction data were collected at beamline PROXIMA1 (SOLEIL synchrotron). The asymmetric unit contains 12 molecules, and EfFIC assemble as hexamers in the crystal. Only the ADP moiety of ATPgS is visible. The structure has been deposited with PDB entry code 6EP2.

For crystals of $EfFIC^{WT}$-ATPγS, the protein was prepared in a buffer containing 50 mM Tris pH 8.0, 100 mM NaCl and 5 mM ATPγS and concentrated to 13 mg/mL. Crystals were obtained in 2 M ammonium sulfate and 0.1 M Bis-Tris pH 5.5. Diffraction data were collected at beamline ID30B (ESRF synchrotron). The asymmetric unit contains 6 molecules, and EfFIC assembles as hexamers in the crystal. Only the ADP moiety of ATPγS is visible. The structure has been deposited with PDB entry code 6EP5.

For crystals of $EfFIC^{H111A}$, the protein was prepared in a buffer containing 50 mM Tris pH 8.0, 200 mM NaCl and 1 mM $MgCl_2$ and concentrated to 10 mg/mL. Crystals were obtained in 0.2 M Ammonium sulfate, 0.1 M Tris-sodium citrate pH 5.6, 15% (w:v) PEG 4000. Diffraction data were collected at beamline PROXIMA2A (SOLEIL synchrotron). The asymmetric unit contains 2 molecules, and EfFIC assembles as tetramers related to the NmFIC tetramer in the crystal. The structure has been deposited with PDB entry code 5NWF.

For crystals of $EfFIC^{H111A}$-SO_4^{2-}, the protein was prepared in a buffer containing 50 mM Tris pH 8.0, 150 mM NaCl, 2 mM ATPγS, and 2 mM $MgCl_2$ and concentrated to 7 mg/mL. Crystals were obtained in 0.2 M lithium Sulfate, 0.1M Tris pH 8.5 and 20% (w/v) PEG 4000. Diffraction data were collected at beamline PROXIMA2A (SOLEIL synchrotron). The asymmetric unit contains 4 molecules, and EfFIC assembles as tetramers related to the NmFIC tetramer. The structure has been deposited with PDB entry code 6ERB.

Supplementary Table 1 : Data collection and refinement statistics.

	**EfFIC^{WT}- PO_4^{2-}**	**EfFIC^{WT}**	**EfFIC^{WT}-AMP-Ca^{2+}**
**Data collection**			
Space group	$P4_12_12$	I222	$P4_12_12$
Cell dimensions			
a, *b*, *c* (Å)	65.13, 65.13, 248.06	121.54, 131.00, 136.94	64.98 64.98 246.24
α, β, γ (°)	90.00, 90.00, 90.00	90.00, 90.00, 90.00	90.00, 90.00, 90.00
Resolution (Å)	44.91-2.29 (2.38-2.29)	47.33-2.40 (2.49-2.40)	82.08-2.35 (2.48-2.35)
R_{merge}	0.312 (1.808)	0.087 (0.665)	0.139 (1.146)
$I / \sigma I$	5.2 (1.4)	13.5 (2.2)	13.1 (2.3)
Completeness (%)	94.8 (91.7)	99.8 (97.6)	100.0 (100.0)
Redundancy	4 (2.8)	5.6 (5.4)	16.7 (17.8)
**Refinement**			
Resolution (Å)	2.29	2.40	2.35
No. reflections	23571	42957	22951
R_{work} / R_{free}	0.229/0.298	0.164/0.226	0.2018/0.264
No. atoms	3653	7315	3528
Protein	3344	6642	3341
Ligand/ion	14	-	54
Water	295	673	133
B-factors	35.0	50.0	61.0
Protein	33.0	46.0	58.0
Ligand/ion	27.9	-	71.5
Water	76.4	52.4	44.3
R.m.s. deviations			
Bond lengths (Å)	0.008	0.010	0.010
Bond angles (°)	0.893	1.08	1.08

Supplementary Table 1, continued

	**EfFIC^{H111A}**	**EfFIC^{H111A} -SO_4^{2-}**
**Data collection**		
Space group	$P2_12 2_1$	I222
Cell dimensions		
a, *b*, *c* (Å)	76.67 77.11 103.15	121.93 131.16 136.71
α, β, γ (°)	90.00, 90.00, 90.00	90.00, 90.00, 90.00
Resolution (Å)	103.15-2.60 (2.72-2.60)	47.32-2.20 (2.26-2.20)
R_{merge}	0.072 (0.499)	0.081 (1.321)
$I / \sigma I$	15.5 (3.6)	18.5 (2.1)
Completeness (%)	99.1 (99.9)	100.0 (100.0)
Redundancy	5.9 (6.1)	13.7 (14.2)
**Refinement**		
Resolution (Å)	2.6	2.2
No. reflections	19240	55796
R_{work} / R_{free}	0.191/0.250	0.2132/0.2488
No. atoms	3514	6918
Protein	3344	6604
Ligand/ion	-	20
Water	170	294
B-factors	62.0	54.0
Protein	55	53
Ligand/ion	-	54.8
Water	41.2	35.3
R.m.s. deviations		
Bond lengths (Å)	0.008	0.008
Bond angles (°)	1.103	1.012

Supplementary Table 1, continued

	EfFIC^{WT}-ATPγS-Ca^{2+}	EfFIC^{WT}- ATPγS
**Data collection**		
Space group	$P4_32_12$	$P4_32_12$
Cell dimensions		
a, *b*, *c* (Å)	125.35 125.35 362.8	87.84 87.84 364.94
α, β, γ (°)	90.00 90.00 90.00	90.00 90.00 90.00
Resolution (Å)	118.45-2.15 (2.19-2.15)	47.3-1.93 (1.96-1.93)
R_{merge}	0.21 (1.952)	0.172 (2.806)
$I / \sigma I$	10.5 (2.1)	9.8 (0.8)
Completeness (%)	100 (100)	99.9 (97.3)
Redundancy	16.9 (17.7)	13.0 (12.6)
**Refinement**		
Resolution (Å)	2.15	1.93
No. reflections	157231	108653
R_{work} / R_{free}	0.1869/0.2178	0.2003/0.2402
No. atoms	22137	11142
Protein	20299	10018
Ligand/ion	348	162
Water	1490	962
B-factors	41.0	33.0
Protein	38.0	32.0
Ligand/ion	42.9	24.5
Water	43.4	41.6
R.m.s. deviations		
Bond lengths (Å)	0.005	0.007
Bond angles (°)	0.947	0.992

Supplementary Table 2: Synthetic genes optimized for *E. coli*

Protein	Gene
$EfFIC^{WT}$	ATGCATCATCATCACCATCACATGCTGGAAAATAAACTGGGCATTATCAATCAGCTGGAACTGAATCGTGTT<br>GAAGAACGTGTTAGCAAAGAAAATGCCAAACGTCTGTATGATAGCGGTGATATTGATCGTATTGAAGTGGG<br>TACATTTAAAGGCCTGAGCTATATTCACAACTACCTGTTCGAAGATATCTACGAGTTTGCAGGTAAAGTTCGC<br>AGCCAGAATATTAGCAAAGGCAATTTTCGTTTTGCACCGGTGATGTATCTGGAAATTGCACTGGAACACATT<br>GATAAAATGCCGCAGCGTAATCTGGATGAAATTGTTGCCAAATACGTGGAAATGAATATCGCACATCCGTT<br>TCGTGAAGGTAATGGTCGTGCAACCCGCATTTGGCTGGATCTGATTCTGAAAAAAGAACTGAAACGCGTGG<br>TTGATTGGAACCTGATTAACAAAGAAGATTATCTGAGCGCCATGGAACGTAGTCCGGTTAAAGACCTGGAAA<br>TCAAATATCTGATTAGCAATGCCCTGACCGACAAAATTAACGATCGTGAGATTTTTATGAAAGGCATCGACAT<br>CAGCTATTATTATGAAGGCTATACCGAGTATAACGTGGATGAACTGTAA
$FICD^{WT}$	AGCGATAGCGAAGTTAATCAAGAAGCCAAACCGGAAGTTAAGCCGGAAGTGAAACCTGAAACACATATTAA<br>CCTGAAAGTGAGTGATGGCAGCAGCGAAATCTTCTTCAAAATCAAAAAAACCACACCGCTGCGTCGTCTGAT<br>GGAAGCATTTGCAAAACGTCAGGGTAAAGAAATGGATAGCCTGCGTTTTCTGTATGATGGTATTCGTATTCA<br>GGCAGATCAGACACCGGAAGATCTGGATATGGAAGATAACGATATTATCGAAGCACATCGTGAGCAGATTG<br>GTGGTAGCGGTGCAGTTGAAGAACAGTGTCTGGCAGTTCTGAAAGGTCTGTATCTGCTGCGTAGCAAACCGG<br>ATCGTGCACAGCATGCAGCAACCAAATGTACCAGTCCGAGCACCGAACTGAGCATTACCAGCCGTGGTGCA<br>ACCCTGCTGGTTGCAAAAACCAAAGCAAGTCCGGCAGGTAAACTGGAAGCACGTGCAGCACTGAATCAGG<br>CACTGGAAATGAAACGCCAGGGCAAACGTGAAAAAGCACAGAAACTGTTTATGCACGCACTGAAAATGGATC<br>CGGATTTTGTTGATGCACTGACCGAATTTGGTATCTTTAGCGAAGAGGATAAAGATATCATCCAGGCCGATT<br>ATCTGTATACCCGTGCGCTGACCATTAGTCCGTATCATGAAAAAGCCCTGGTTAATCGTGATCGTACCCTGCC<br>GCTGGTTGAAGAAATTGATCAGCGTTATTTCAGCATCATCGACAGCAAAGTCAAAAAGGTTATGAGCATTCC<br>GAAAGGTAATAGCGCACTGCGTCGCGTTATGGAAGAAACCTACTATCATCATATCTATCACACCGTTGCCATT<br>GAAGGTAATACCCTGACACTGAGCGAAATTCGTCATATTCTGGAAACCCGTTATGCAGTTCCGGGTAAAAGCC<br>TGGAAGAACAGAATGAAGTTATTGGTATGCATGCAGCCATGAAGTATATTAACACCACACTGGTTAGCCGTA<br>TTGGTAGCGTTACCATTAGTGATGTTCTGGAAATTCATCGTCGTGTTCTGGGTTATGTTGATCCGGTTGAAGC<br>AGGTCGTTTTCGTACCACACAGGTTCTGGTTGGTCATCATATTCCGCCTCATCCGCAGGATGTTGAAAAACAA<br>ATGCAAGAATTTGTGCAGTGGCTGAATAGCGAAGAAGCAATGAATCTGCATCCGGTGGAATTTGCAGCCCTG<br>GCACATTATAAACTGGTTTATATTCACCCGTTCATCGATGGTAATGGTCGTACCAGTCGTCTGCTGATGAATCT<br>GATTCTGATGCAGGCAGGTTATCCGCCTATTACCATTCGTAAAGAACAGCGCAGCGATTATTATCATGTGCTG<br>GAAGCAGCAAATGAAGGTGATGTTCGTCCGTTTATTCGCTTTATTGCAAAATGTACCGAAACCACGCTGGATA<br>CACTGCTGTTTGCCACCACCGAATATAGCGTTGCACTGCCGGAAGCACAGCCGAATCATAGCGGTTTTAAAGA<br>AACCCTGCCGGTTAAACCGTAA

Supplementary Table 3: Primers used in the study

Protein	Primer Forward	Primer Reverse
EfFIC^{E115A}	CGCACATCCGTTTCGTGCAGGTAATGGTCGTGCAACCC	GGGTTGCACGACCATTACCTGCACGAAACGGATGTGCG
EfFIC^{E190G}	CGACATCAGCTATTATTATGGAGGCTATACCGAG	CTCGGTATAGCCTCCATAATAATAGCTGATGTCG
FICD^{E234G}	CACACCGTTGCCATTGGAGGTAATACCCTGACACTG	CAGTGTCAGGGTATTACCTCCAATGGCAACGGTGTG

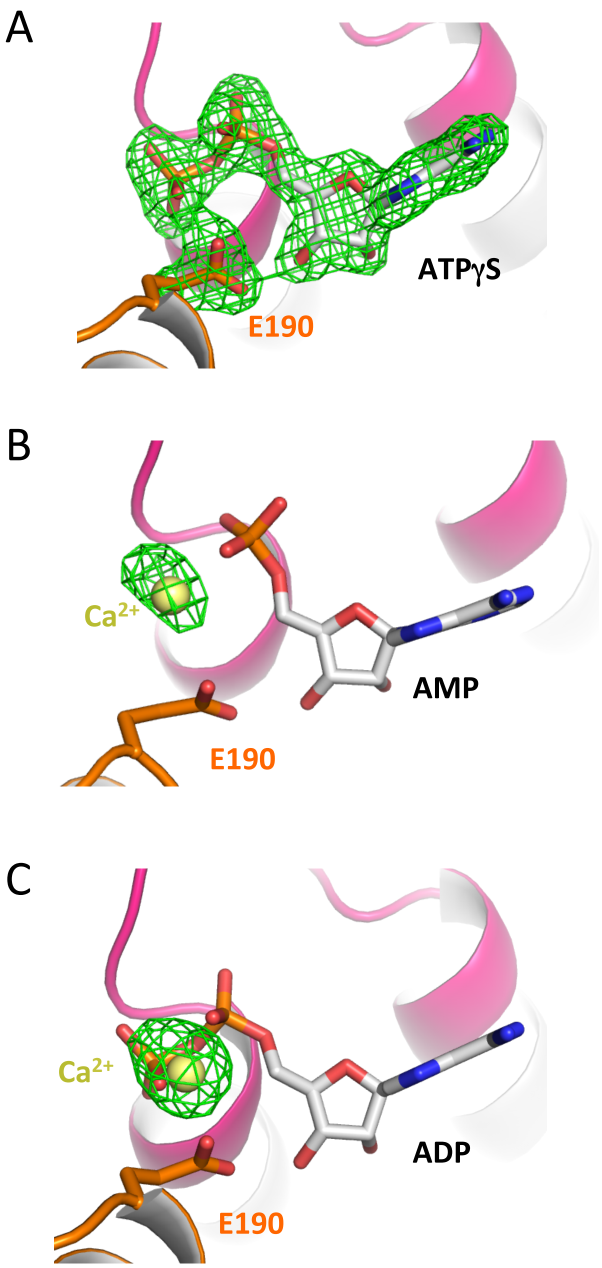

Supplementary Figure 1. Structural analysis of EfFIC.
A: Omit map showing the electron density of the inhibitory glutamate (E190) and the ADP moiety of ATPγS in $EfFIC^{WT}$-ATPγS contoured at 5.0 σ. The electron density of Glu190 is representative of all EfFIC structures determined in this study.
B: Omit map showing the electron density of Ca^{2+} in $EfFIC^{WT}$-AMP-Ca^{2+}-bound structure contoured at 4.0 σ.
C: Omit map showing the electron density of Ca^{2+} in $EfFIC^{WT}$-ATPγS-Ca^{2+}-bound structure contoured at 5.0 σ.

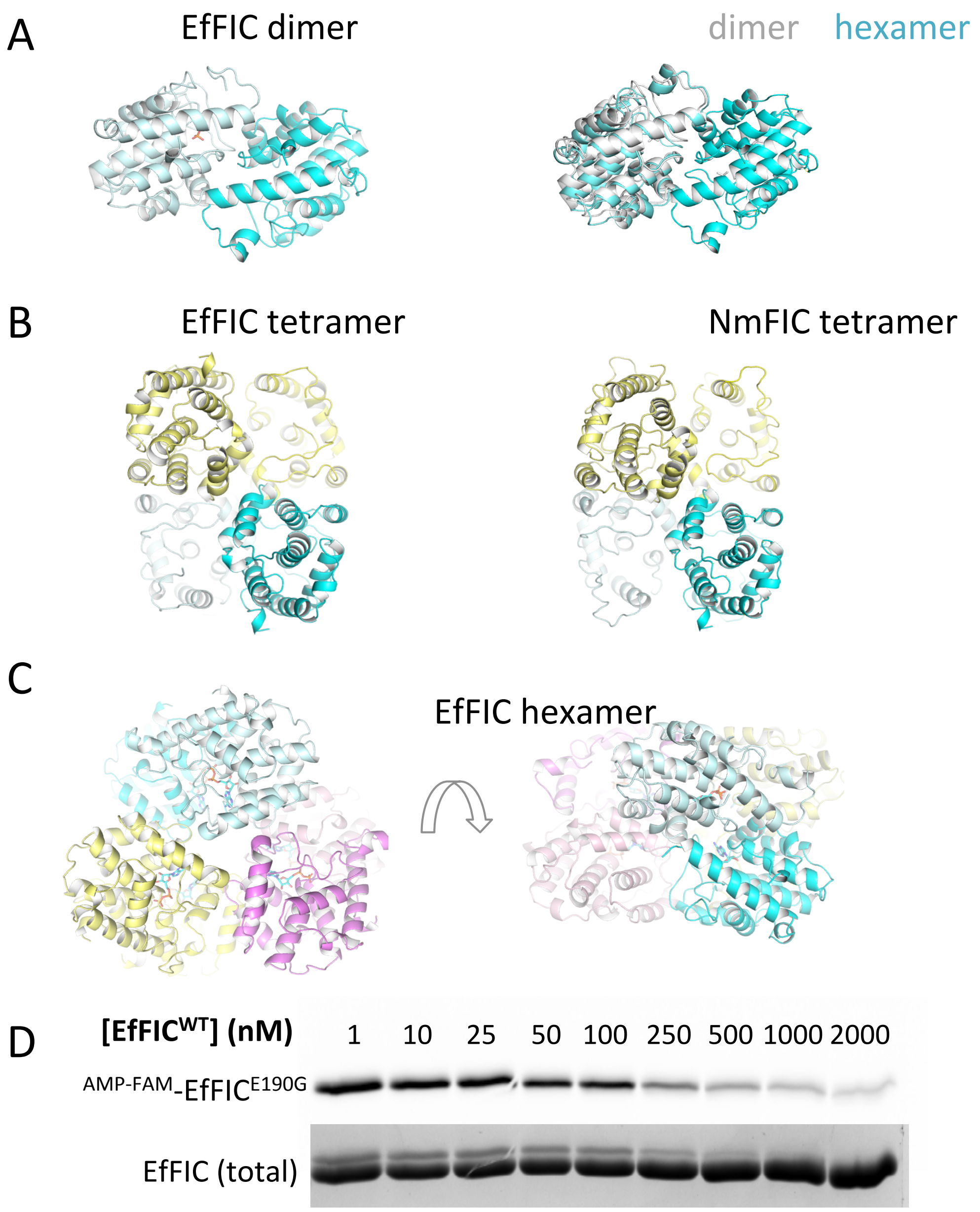

Supplementary Figure 2 - Analysis of EfFIC oligomeric assemblies.
The oligomeric assemblies observed in each crystal form are given in **Supplementary Information**.
A: EfFIC forms equivalent dimers in all crystal forms. Left: The two monomers are shown in two shades of blue. Right: Superposition of EfFIC dimers in dimeric (grey) and hexameric (cyan) assemblies, showing slight differences in the dimeric arragement. The superposition is done on one monomer.
B: EfFIC forms tetramers related to the NmFIC tetramer)[4] in some crystal forms. The dimers are highlighted in shades of yellow and blue.
C: EfFIC forms hexamers in some crystal forms, shown in two orthogonal views. The dimers are highlighted in shades of yellow, blue and pink.
D: DeAMPylation is not inhibited at high EfFIC concentration. Purified $^{AMP-FAM}$EfFIC^{E190G} was incubated with increasing concentrations of EfFIC^{WT} for one hour in the presence of 100 μM Ca^{2+}. AMPylation levels were measured by fluorescence as in **Figure 2D**.

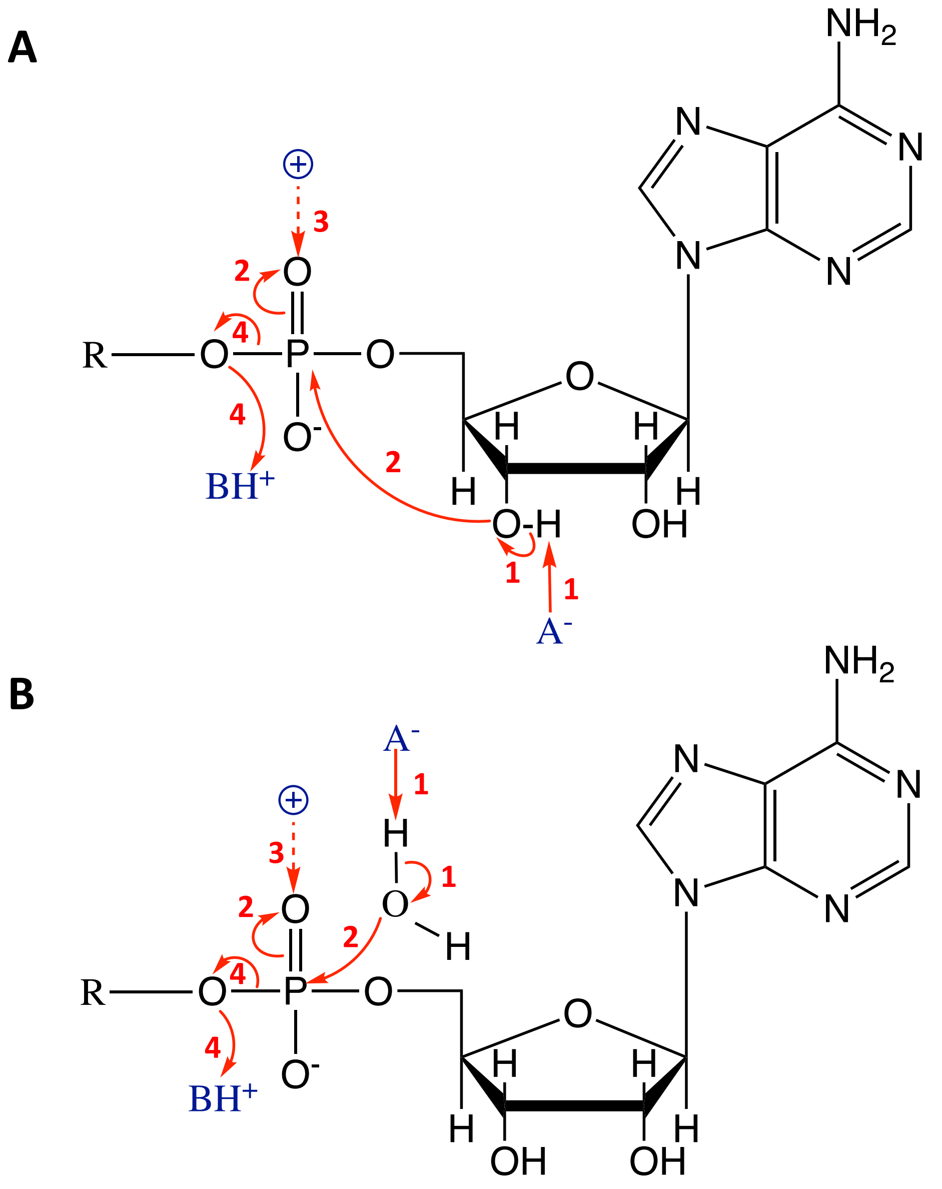

Supplementary Figure 3. Comparison of anchimeric and acido-basic deAMPylation catalytic mechanisms.

A: Anchimeric catalysis.

B: Acido-basic catalysis.

Both mechanisms involve four steps, as indicated: (1) activation of the reactive oxygen through proton attraction (2); nucleophilic attack on the positively charged phosphorus triggering P=O p electrons rearrangement and production of a pentavalent intermediate harboring an additional negative charge; (3) stabilization of the intermediate by a positive charge in the catalytic site, which also contribute to elicit the electrophily of the phosphorus and (4) facilitation of phosphor-ester bond cleavage through protonation of the leaving hydroxylate group. R: AMPylated protein. A- : basic form of an acidic catalyst (proton attractor). BH+ : acidic form of a basic catalyst (proton donor).

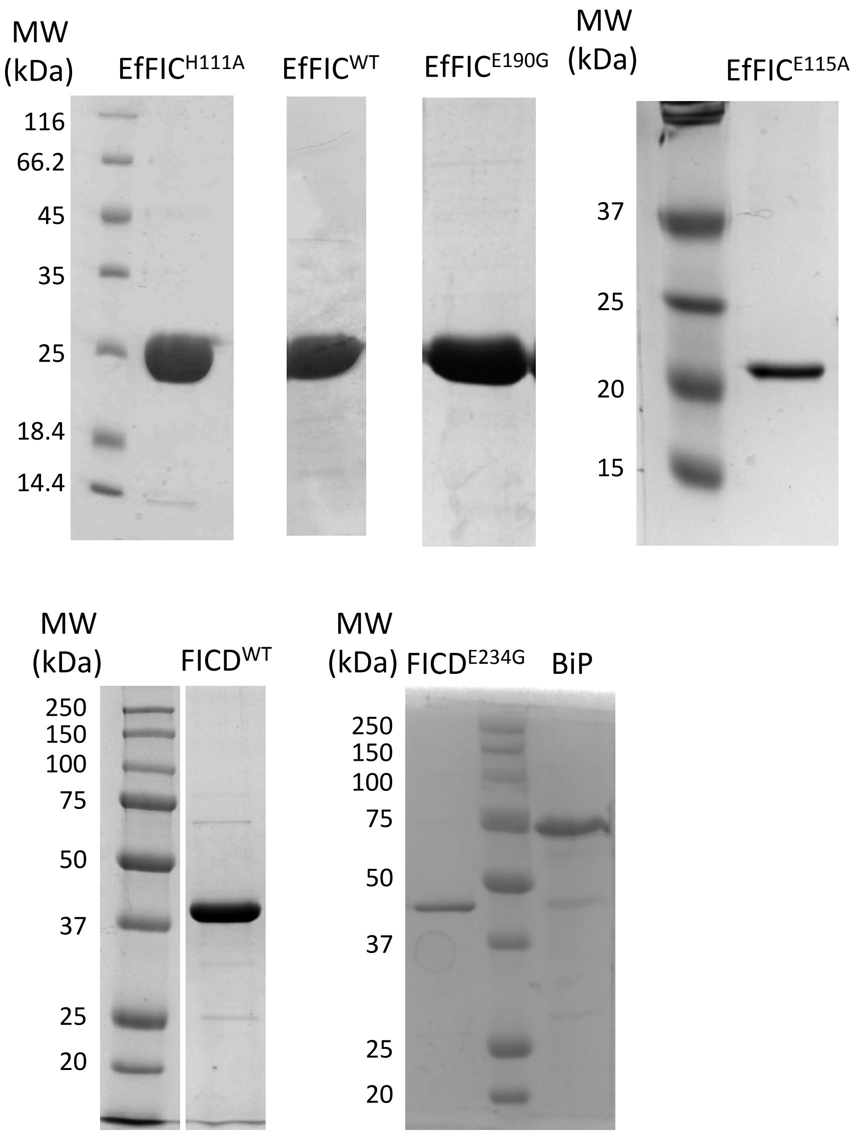

Supplementary Figure 4. Purity of proteins used in this study, analyzed by SDS-PAGE and Coomassie staining

Supplementary References

1. Chen Y*, et al.* Structural basis for Rab1 de-AMPylation by the Legionella pneumophila effector SidD. *PLoS pathogens* **9**, e1003382 (2013).

2. Xu Y, Carr PD, Vasudevan SG, Ollis DL. Structure of the adenylylation domain of E. coli glutamine synthetase adenylyl transferase: evidence for gene duplication and evolution of a new active site. *J Mol Biol* **396**, 773-784 (2010).

3. Moroz OV*, et al.* The structure of a calcium-dependent phosphoinositide-specific phospholipase C from Pseudomonas sp. 62186, the first from a Gram-negative bacterium. *Acta Crystallogr D Struct Biol* **73**, 32-44 (2017).

4. Stanger FV*, et al.* Intrinsic regulation of FIC-domain AMP-transferases by oligomerization and automodification. *Proceedings of the National Academy of Sciences of the United States of America* **113**, E529-537 (2016).
