# Supplementary material for: A Ca2+-regulated deAMPylation switch in human and bacterial FIC proteins
Source: Nat Commun. 2019 Mar 8;10:1142. doi: 10.1038/s41467-019-09023-1 (PMC6408439; doi:10.1038/s41467-019-09023-1)
Supplement: Supplementary file 3 — Reporting Summary [file 41467_2019_9023_MOESM3_ESM.pdf]

nature research

Corresponding author(s): Jacqueline CHERFILS

Last updated by author(s): Jan 11, 2019

Reporting Summary

Nature Research wishes to improve the reproducibility of the work that we publish. This form provides structure for consistency and transparency in reporting. For further information on Nature Research policies, see Authors & Referees and the Editorial Policy Checklist.

Statistics

For all statistical analyses, confirm that the following items are present in the figure legend, table legend, main text, or Methods section.

n/a	Confirmed	
☐	☒	The exact sample size (*n*) for each experimental group/condition, given as a discrete number and unit of measurement
☐	☒	A statement on whether measurements were taken from distinct samples or whether the same sample was measured repeatedly
☐	☒	The statistical test(s) used AND whether they are one- or two-sided<br>*Only common tests should be described solely by name; describe more complex techniques in the Methods section.*
☒	☐	A description of all covariates tested
☐	☒	A description of any assumptions or corrections, such as tests of normality and adjustment for multiple comparisons
☐	☒	A full description of the statistical parameters including central tendency (e.g. means) or other basic estimates (e.g. regression coefficient) AND variation (e.g. standard deviation) or associated estimates of uncertainty (e.g. confidence intervals)
☐	☒	For null hypothesis testing, the test statistic (e.g. *F*, *t*, *r*) with confidence intervals, effect sizes, degrees of freedom and *P* value noted<br>*Give P values as exact values whenever suitable.*
☒	☐	For Bayesian analysis, information on the choice of priors and Markov chain Monte Carlo settings
☒	☐	For hierarchical and complex designs, identification of the appropriate level for tests and full reporting of outcomes
☒	☐	Estimates of effect sizes (e.g. Cohen's *d*, Pearson's *r*), indicating how they were calculated

Our web collection on statistics for biologists contains articles on many of the points above.

Software and code

Policy information about availability of computer code

Data collection	Gels were imaged and quantified using Image lab v6.0. Crystallographic data were collected on synchrotron beamline software
Data analysis	Histograms were plotted with Microsoft Excel v16.9 ; Graphs were plotted in Microsoft Excel v16.9 or GraphPad Prism v7.0; kinetics were analyzed using GraphPad Prism v7.0; Diffraction data were processed with autoPROC or xdsme ; Molecular replacement was performed with Phaser from the Phenix and refinements were performed using Phenix, Buster and Coot; Gels were imaged and quantified using Image lab v6.0. Superpositions of crystallographic structures were performed with Pymol v2.2.0 ; Molecular model drawing was perfomed with ChemDraw Professional v16.0 ; Statistics were performed with XLSTAT v2018.6

For manuscripts utilizing custom algorithms or software that are central to the research but not yet described in published literature, software must be made available to editors/reviewers. We strongly encourage code deposition in a community repository (e.g. GitHub). See the Nature Research guidelines for submitting code & software for further information.

Data

Policy information about availability of data

All manuscripts must include a data availability statement. This statement should provide the following information, where applicable:

- Accession codes, unique identifiers, or web links for publicly available datasets
- A list of figures that have associated raw data
- A description of any restrictions on data availability

Coordinates and structure factors have been deposited in the Protein Data Bank under accession codes 6ER6,5NV5,6EP0,6EP2,6ERB,5NWF,6EP5. The uncropped gels underlying Figs 1F, 2D-E, 3A, 3C-D, 4A-C and the reported averages in the graphs of Figs 3A, 3D and 4C are provided as a Source Data file. A reporting summary for this Article is available as a Supplementary Information file. All other data supporting the findings of this study are available from the corresponding author on reasonable request.

Field-specific reporting

Please select the one below that is the best fit for your research. If you are not sure, read the appropriate sections before making your selection.

☒ Life sciences ☐ Behavioural & social sciences ☐ Ecological, evolutionary & environmental sciences

For a reference copy of the document with all sections, see nature.com/documents/nr-reporting-summary-flat.pdf

Life sciences study design

All studies must disclose on these points even when the disclosure is negative.

Sample size	The chosen sample sizes produced suitable standard deviations.
Data exclusions	Data exclusion in crystallographic datasets (outlier reflection rejection) was carried out automatically as implemented in the program xdsme or autoProc using pre-established criteria.<br>No biochemical data were excluded from the analyses.
Replication	AMPylation assays were performed using different batch of purified protein, substrate (ATP-FAM or radioactive ATP) and buffer every two experiments. Results were highly consistent between each batches. All crystallization conditions have been reproduced with different batches of crystallization buffers.
Randomization	Randomization is not relevant in this study as is does not contain any in vivo studies.
Blinding	Blinding is not relevant in this study as is does not contain any in vivo studies.

Reporting for specific materials, systems and methods

We require information from authors about some types of materials, experimental systems and methods used in many studies. Here, indicate whether each material, system or method listed is relevant to your study. If you are not sure if a list item applies to your research, read the appropriate section before selecting a response.

Materials & experimental systems

n/a	Involved in the study	
☒	☐	Antibodies
☒	☐	Eukaryotic cell lines
☒	☐	Palaeontology
☒	☐	Animals and other organisms
☒	☐	Human research participants
☒	☐	Clinical data

Methods

n/a	Involved in the study	
☒	☐	ChIP-seq
☒	☐	Flow cytometry
☒	☐	MRI-based neuroimaging
